# Supplementary material for: Identification of a weight loss-associated causal eQTL in MTIF3 and the effects of MTIF3 deficiency on human adipocyte function
Source: eLife. 2023 Mar 6;12:e84168. doi: 10.7554/eLife.84168 (PMC10023155; doi:10.7554/eLife.84168)
Supplement: Figure 3—source data 1. [file elife-84168-fig3-data1.zip › Figure 3-source data 1.pptx]

## Slide 1
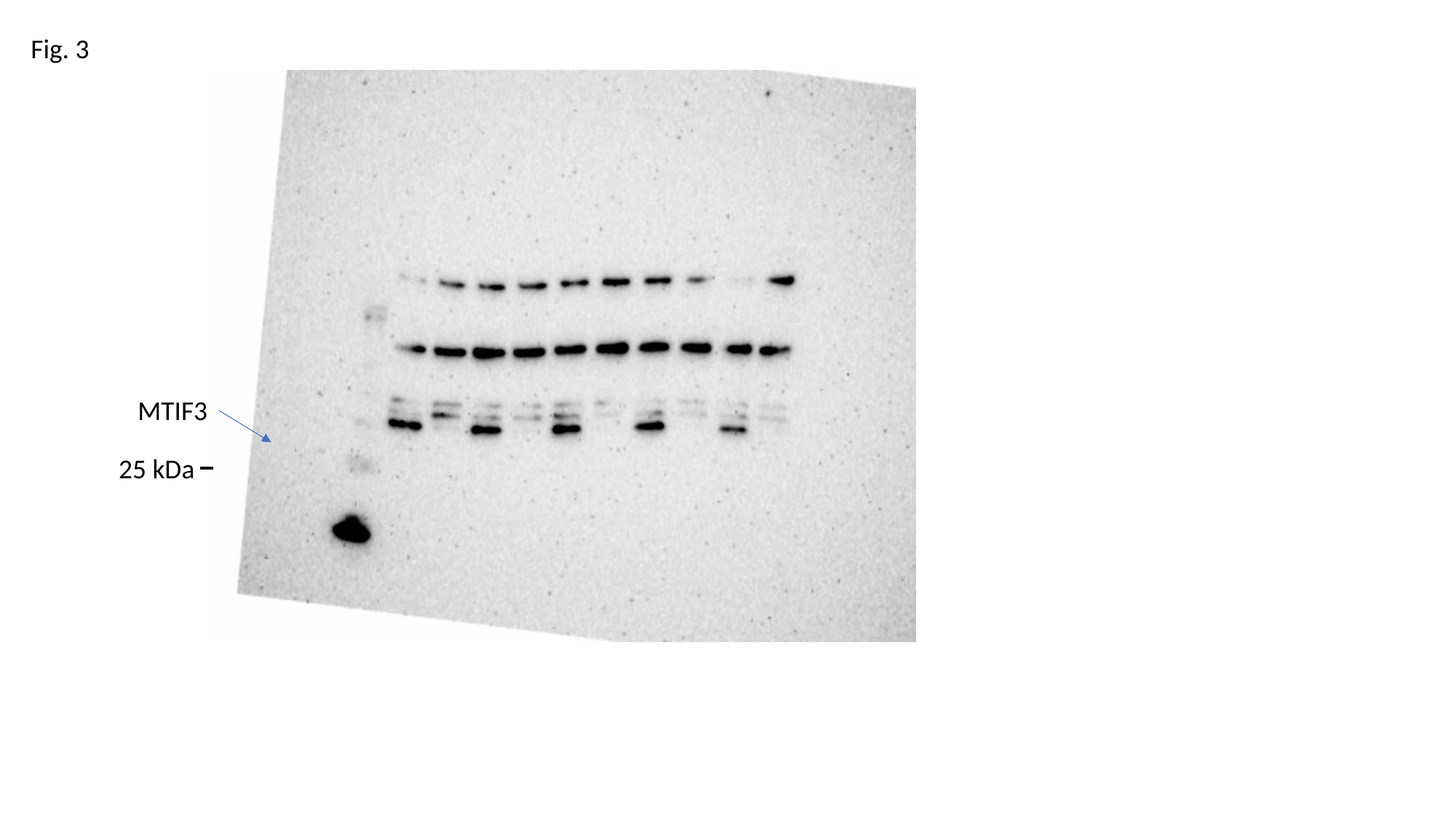

Fig. 3
MTIF3
25 kDa

## Slide 2
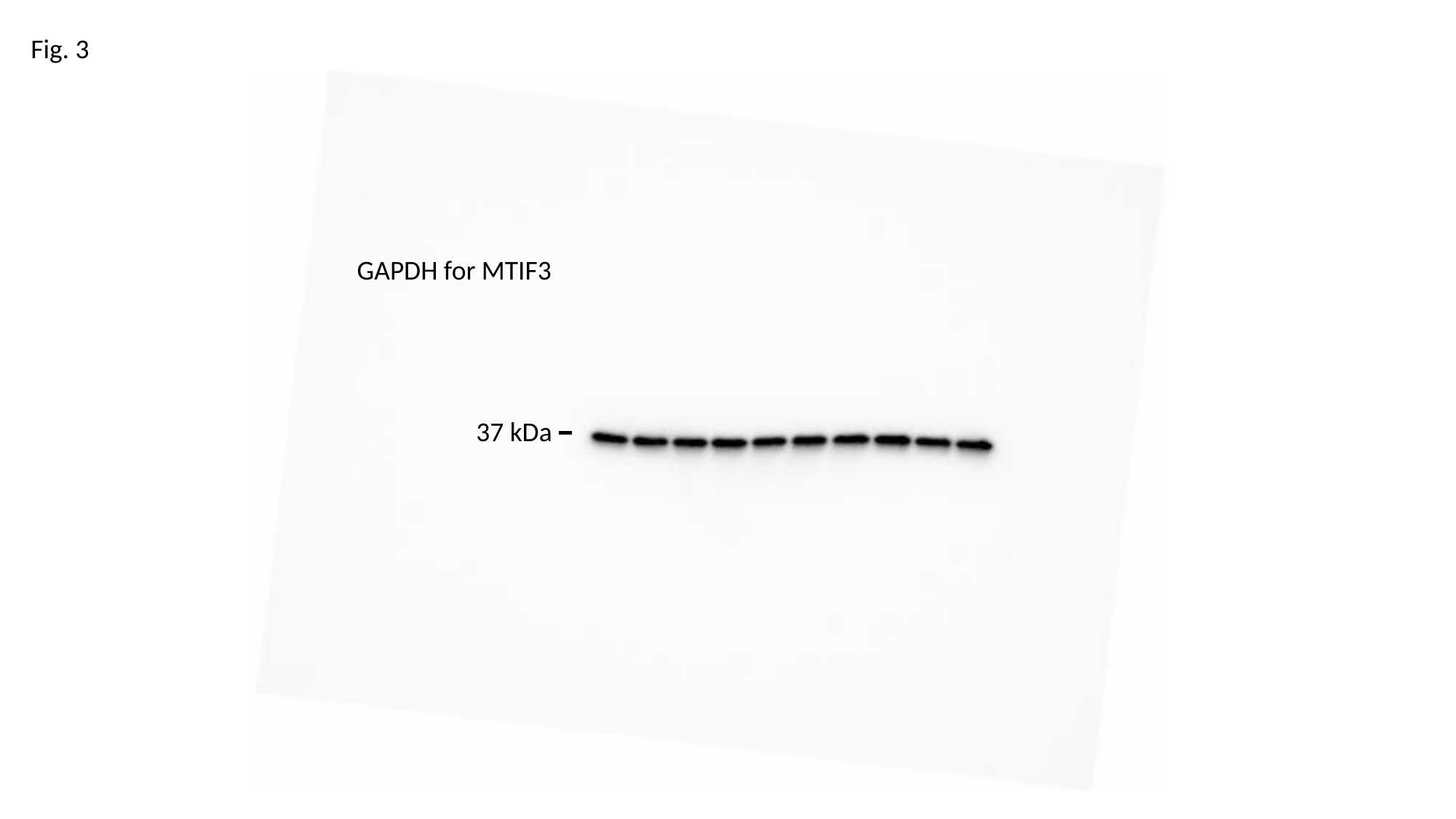

Fig. 3
GAPDH for MTIF3
37 kDa

## Slide 3
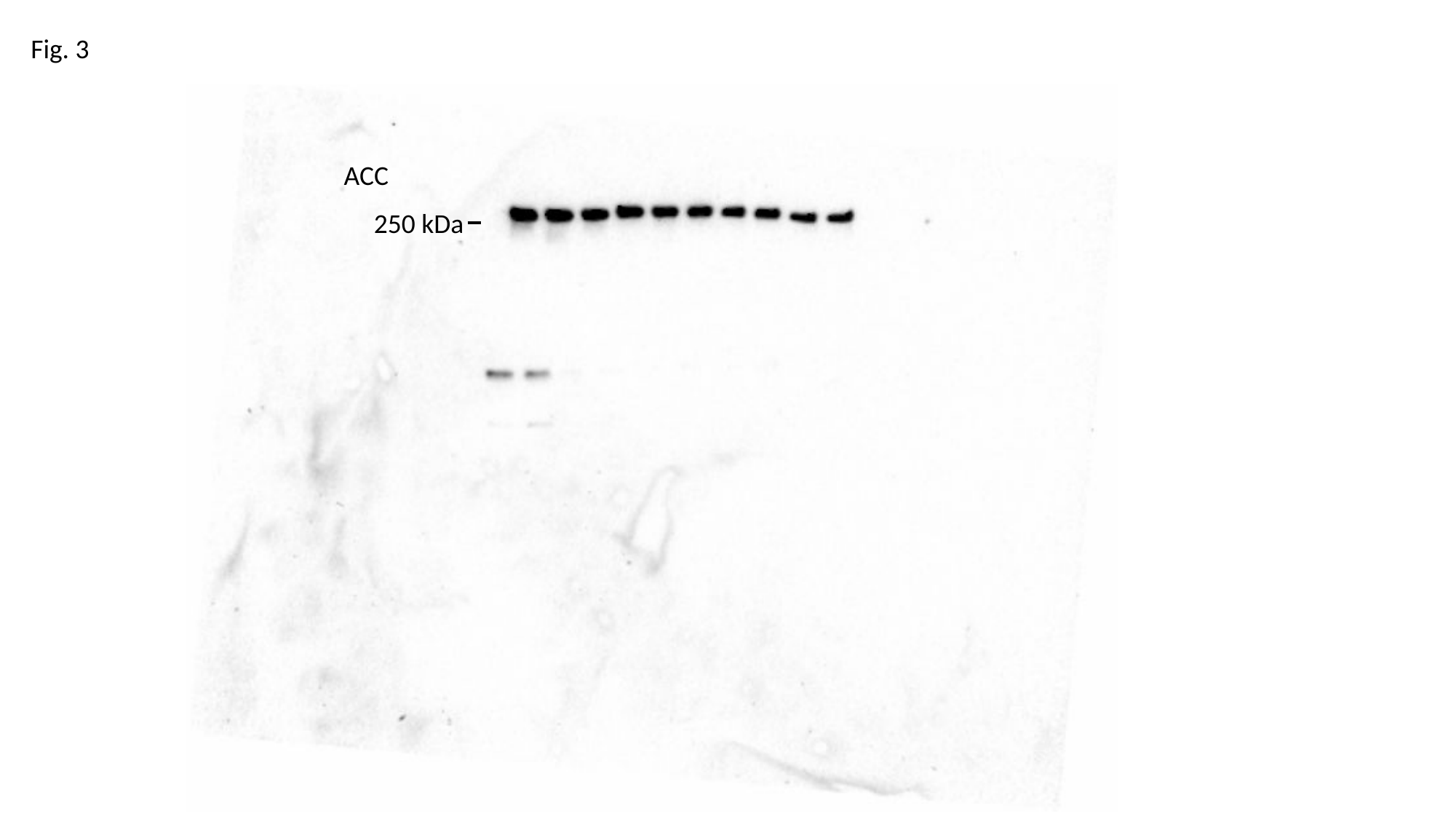

Fig. 3
ACC
250 kDa

## Slide 4
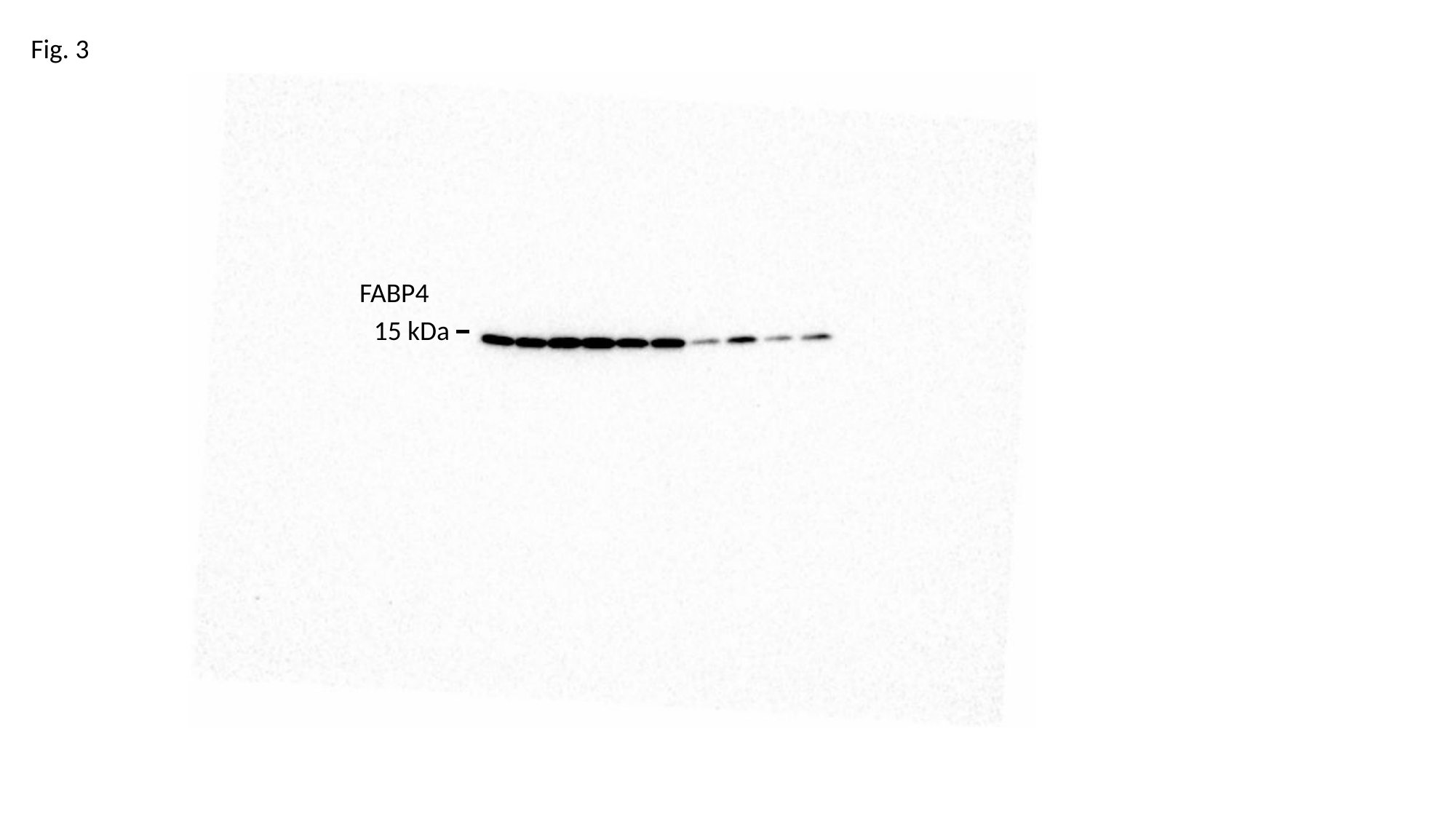

Fig. 3
FABP4
15 kDa

## Slide 5
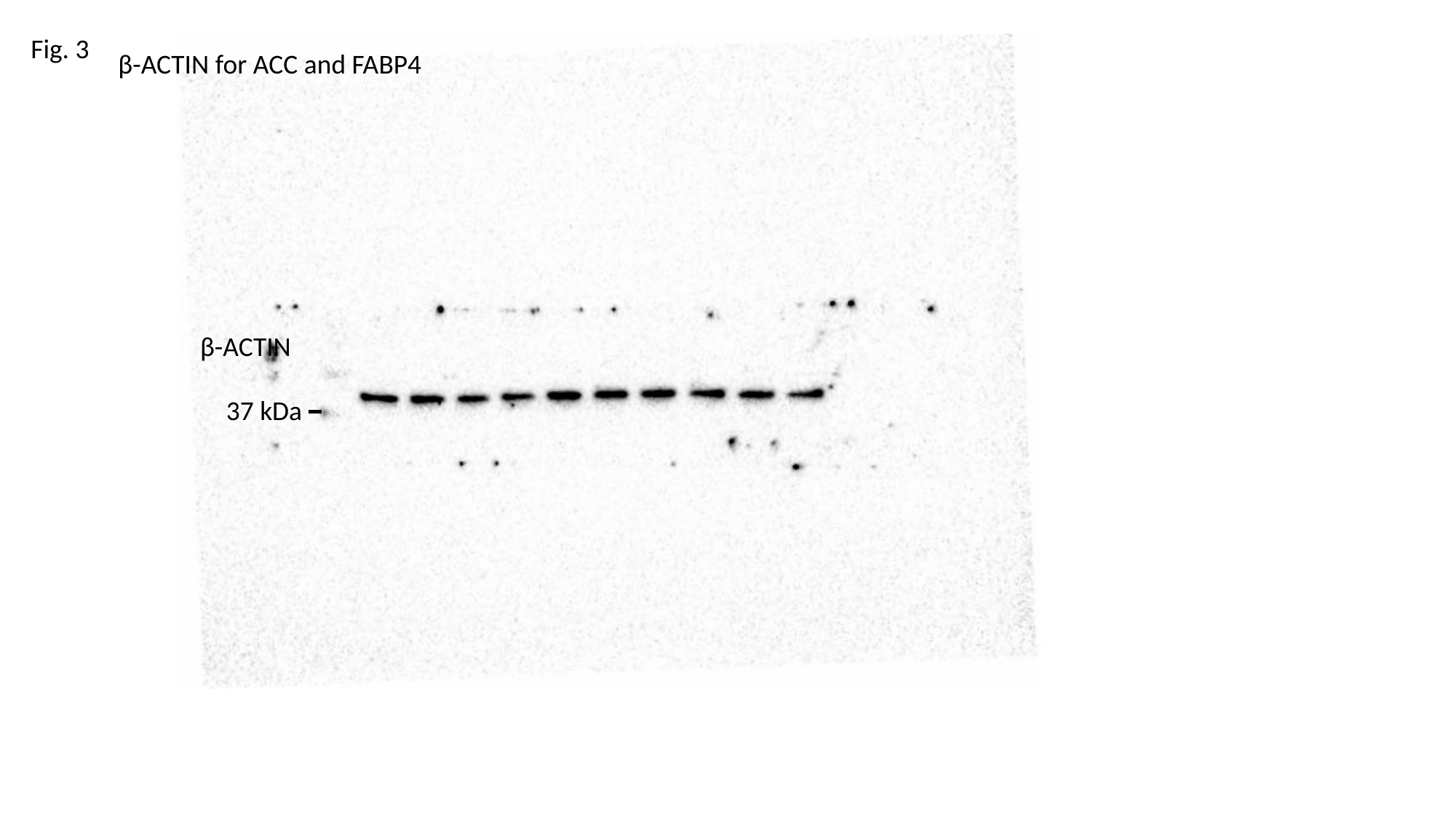

Fig. 3
β-ACTIN for ACC and FABP4
β-ACTIN
37 kDa

## Slide 6
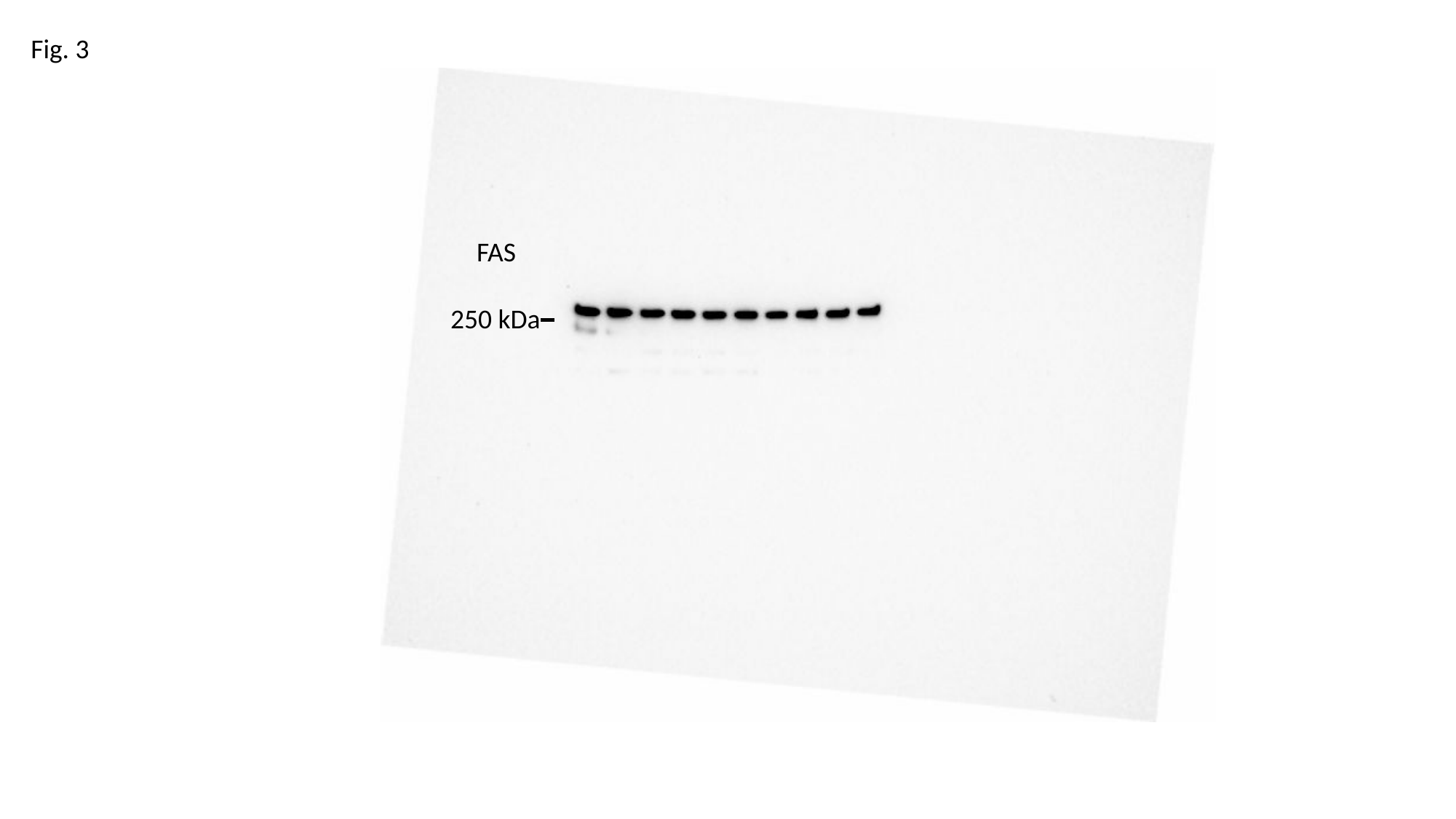

Fig. 3
FAS
250 kDa

## Slide 7
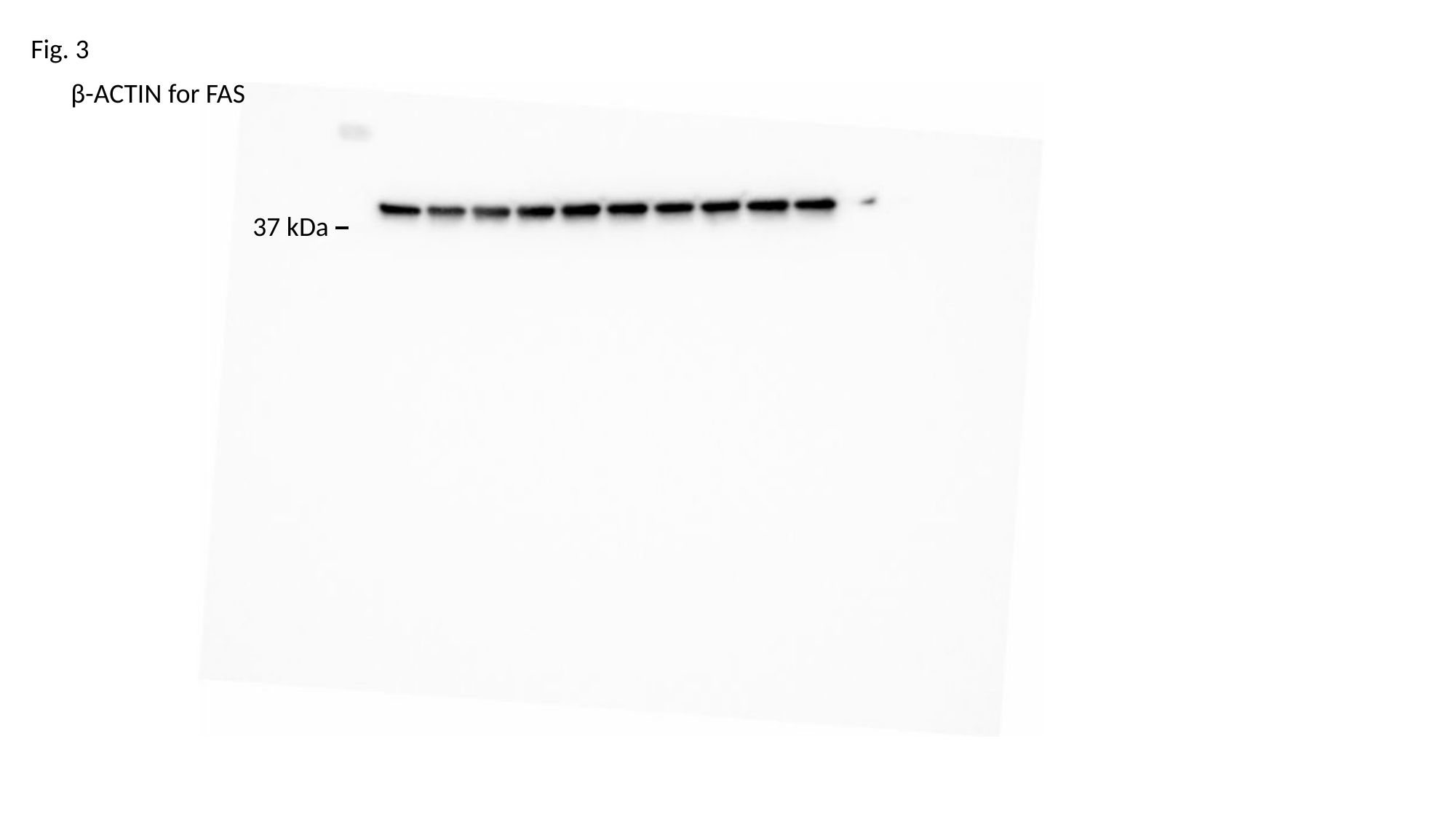

Fig. 3
β-ACTIN for FAS
37 kDa
