# Supplementary material for: Identification of a weight loss-associated causal eQTL in MTIF3 and the effects of MTIF3 deficiency on human adipocyte function
Source: eLife. 2023 Mar 6;12:e84168. doi: 10.7554/eLife.84168 (PMC10023155; doi:10.7554/eLife.84168)
Supplement: Figure 3—figure supplement 1—source data 1. [file elife-84168-fig3-figsupp1-data1.zip › Figure 3-figure supplement 1-uncropped blots.pptx]

## Slide 1
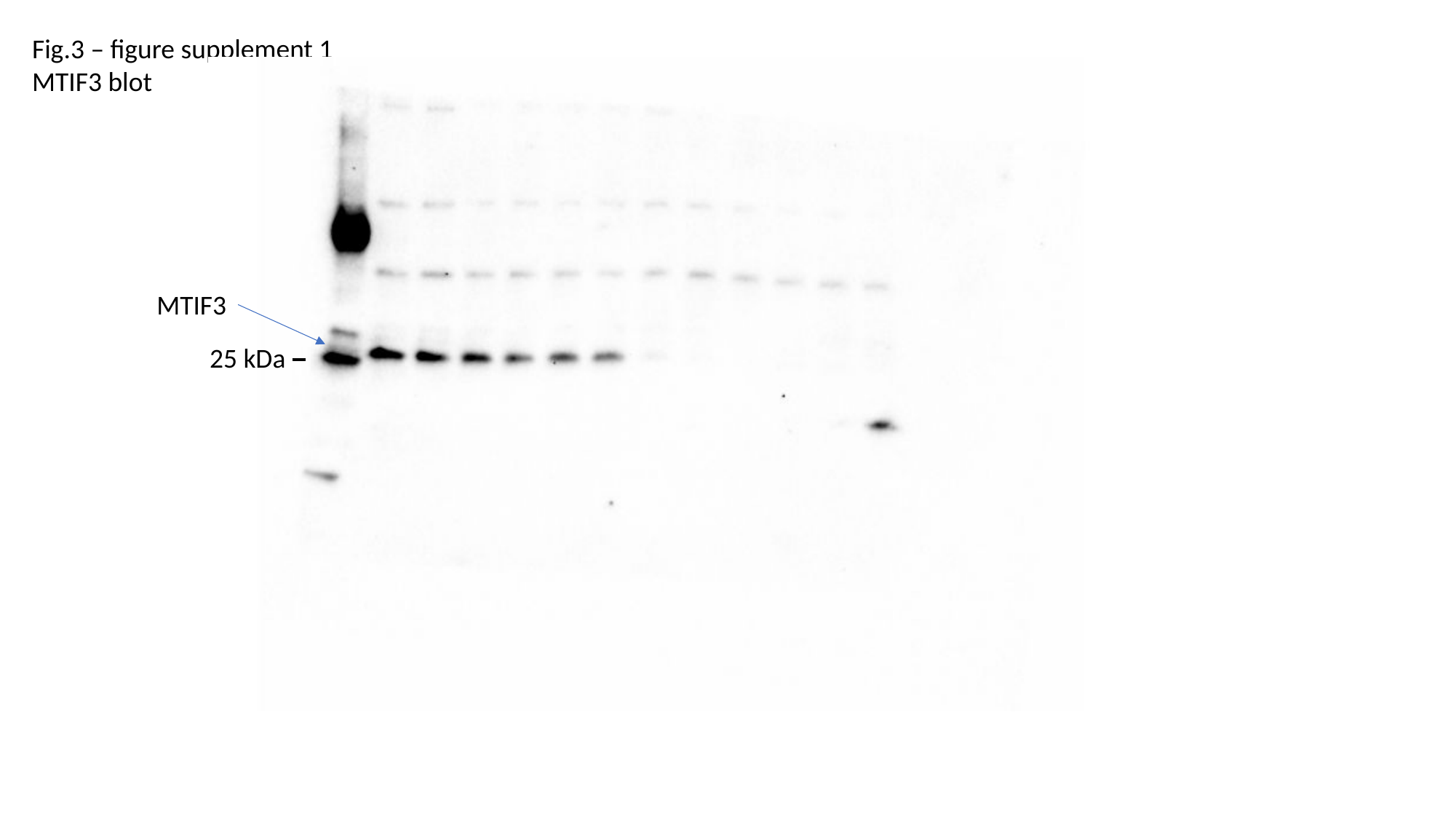

Fig.3 – figure supplement 1
MTIF3 blot
MTIF3
25 kDa

## Slide 2
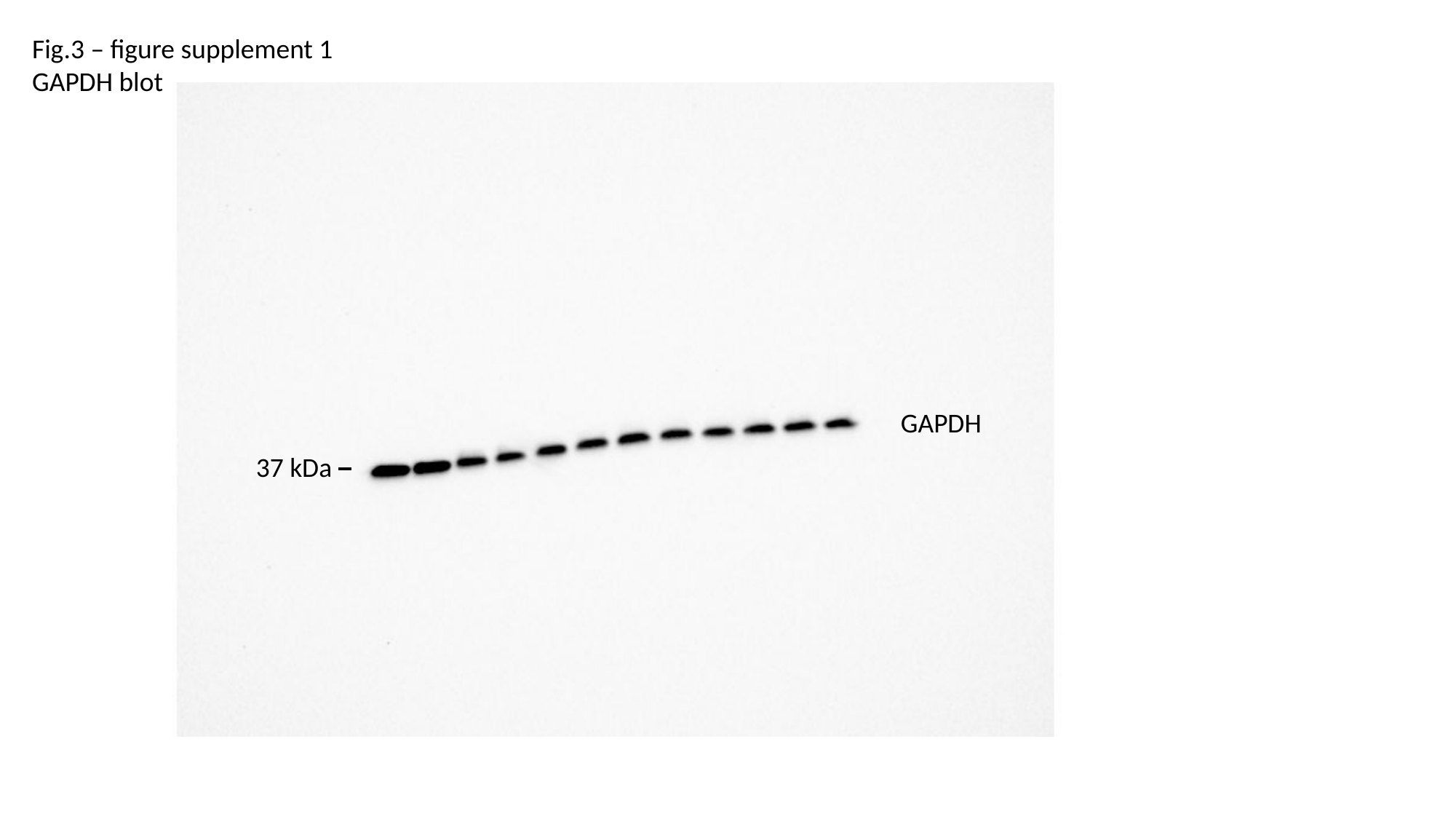

Fig.3 – figure supplement 1
GAPDH blot
GAPDH
37 kDa
