# Supplementary material for: Identification of a weight loss-associated causal eQTL in MTIF3 and the effects of MTIF3 deficiency on human adipocyte function
Source: eLife. 2023 Mar 6;12:e84168. doi: 10.7554/eLife.84168 (PMC10023155; doi:10.7554/eLife.84168)
Supplement: Figure 4—source data 1. [file elife-84168-fig4-data1.zip › Figure 4-source data 1.pptx]

## Slide 1
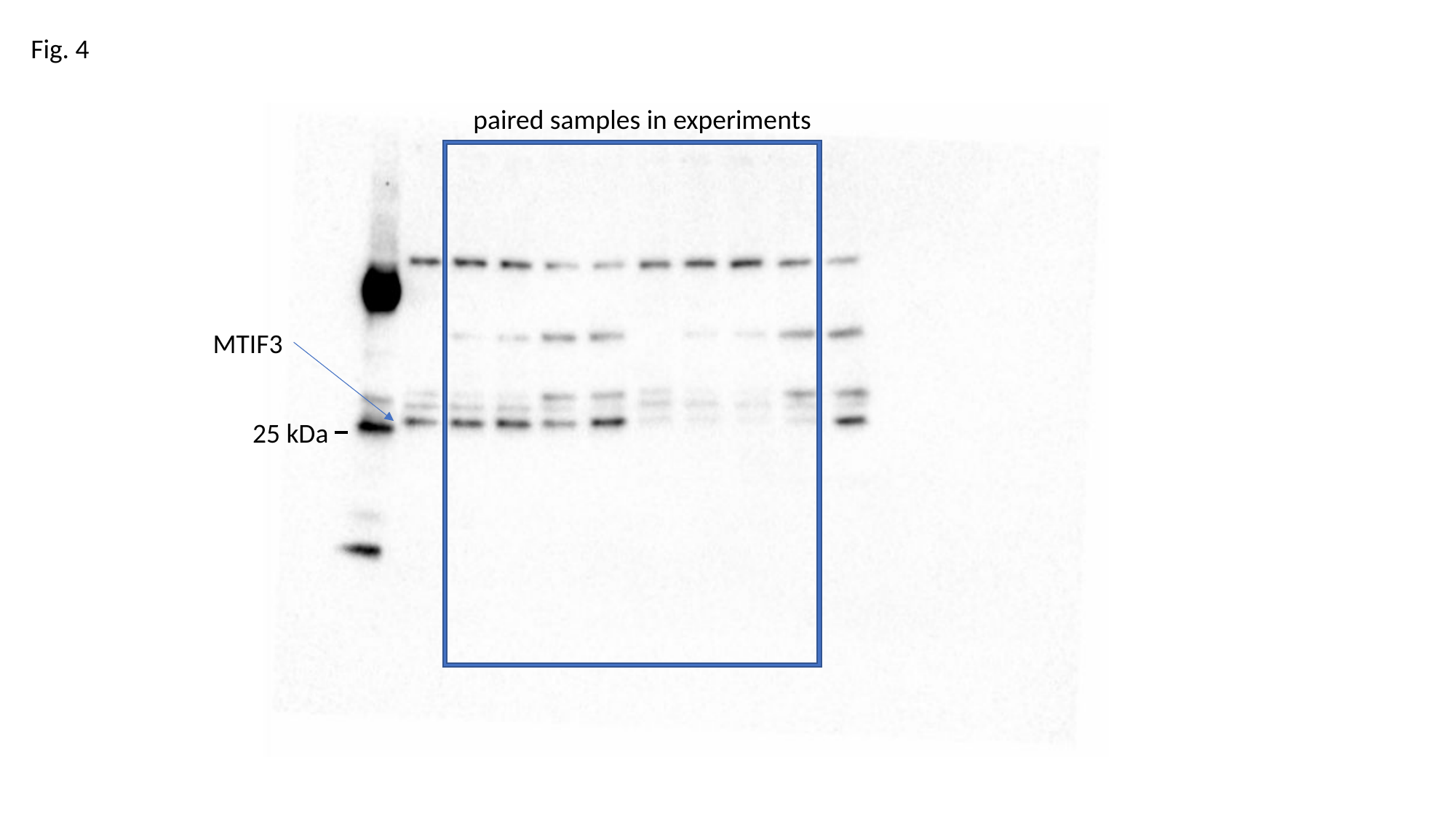

Fig. 4
paired samples in experiments
MTIF3
25 kDa

## Slide 2
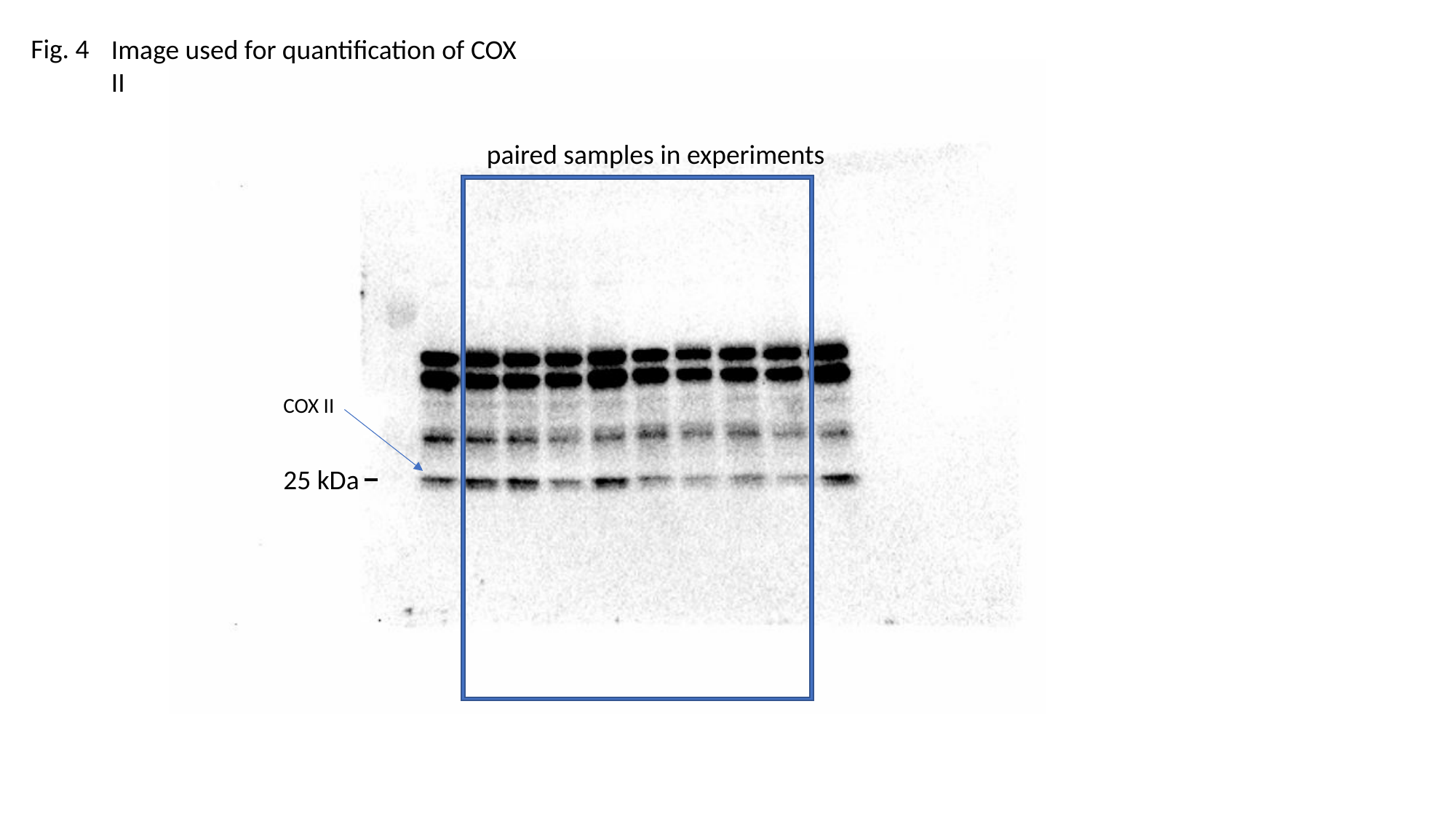

Fig. 4
Image used for quantification of COX II
paired samples in experiments
COX II
25 kDa

## Slide 3
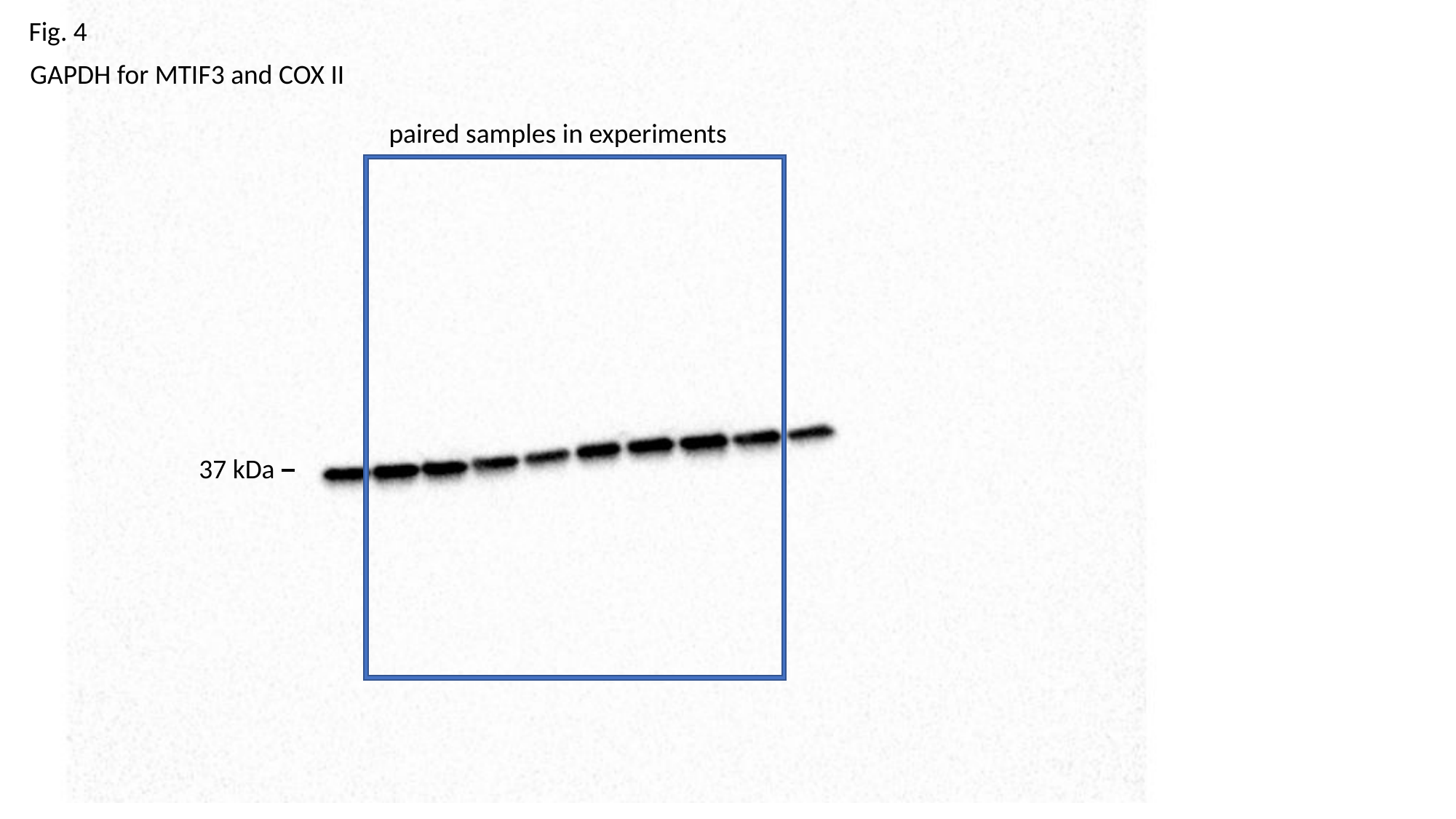

Fig. 4
GAPDH for MTIF3 and COX II
paired samples in experiments
37 kDa

## Slide 4
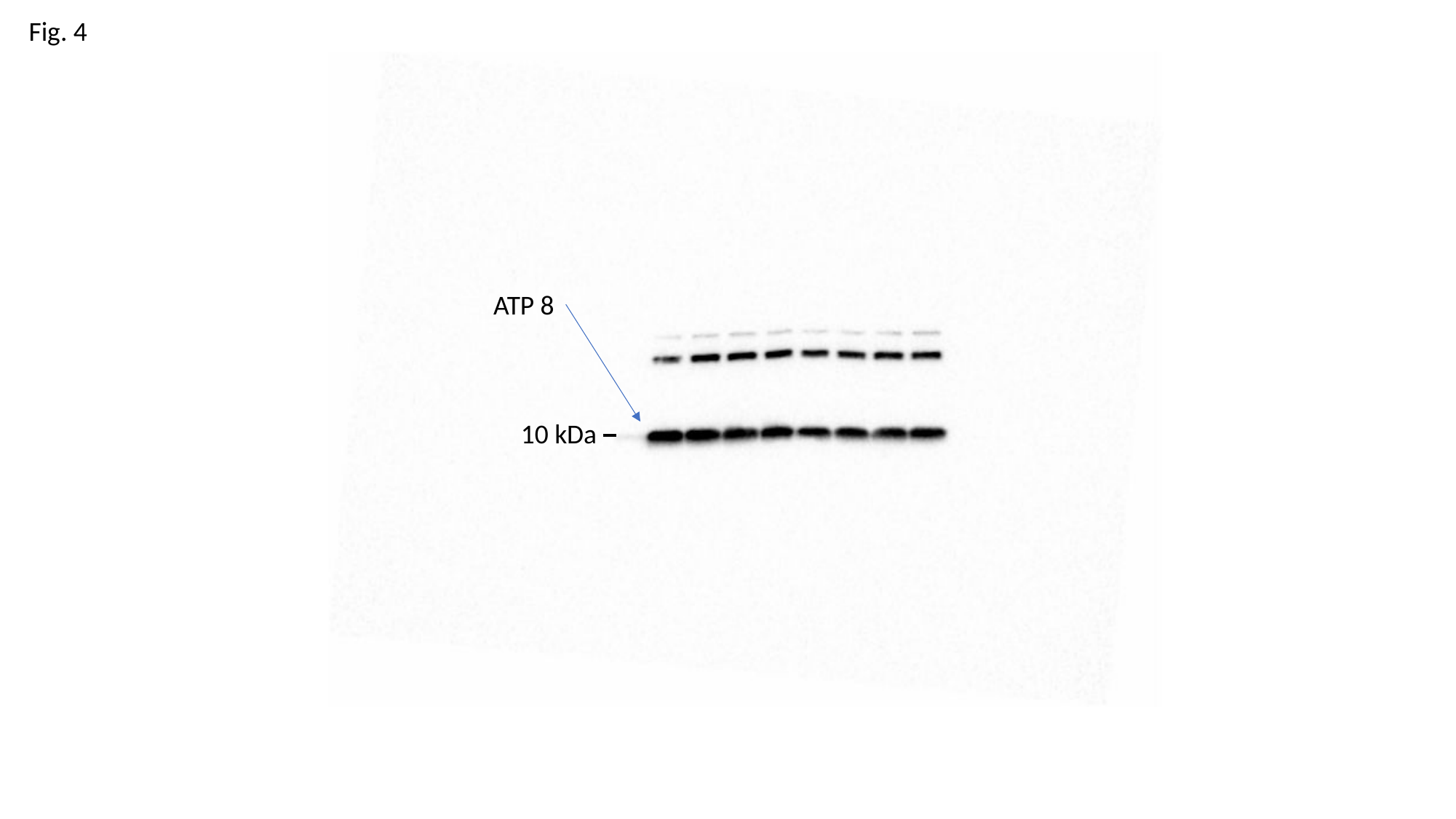

Fig. 4
ATP 8
10 kDa

## Slide 5
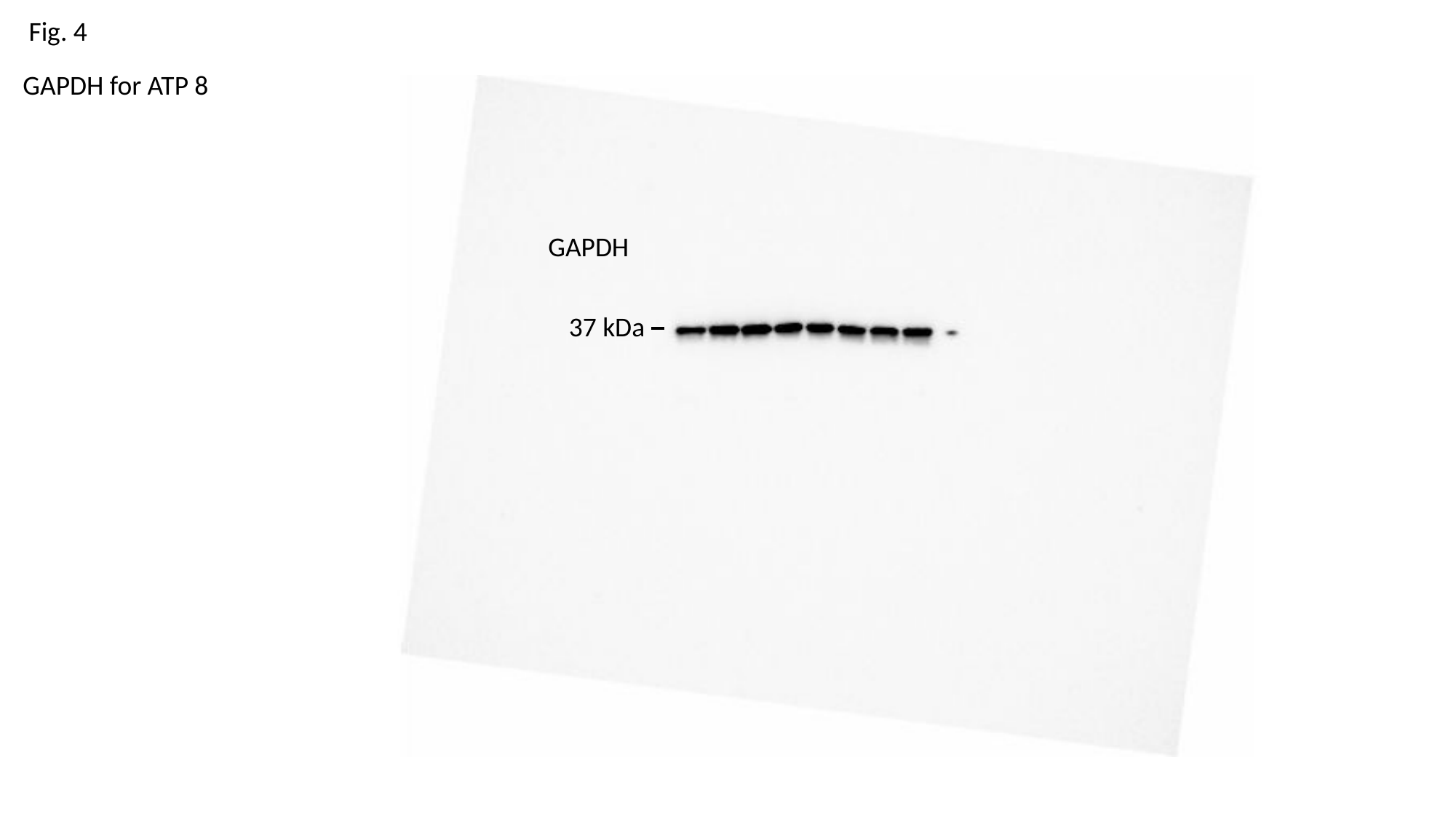

Fig. 4
GAPDH for ATP 8
GAPDH
37 kDa

## Slide 6
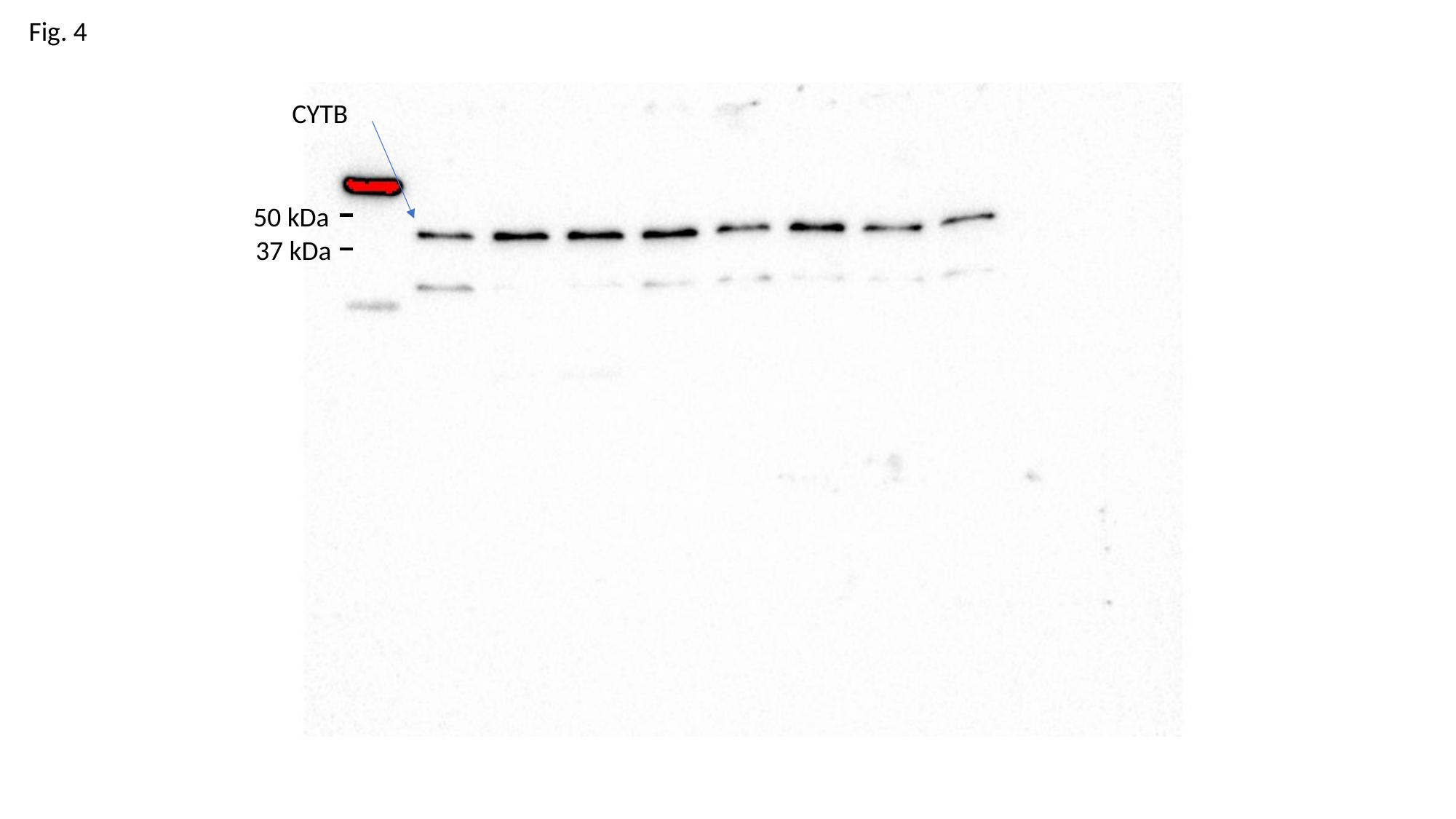

Fig. 4
CYTB
50 kDa
37 kDa

## Slide 7
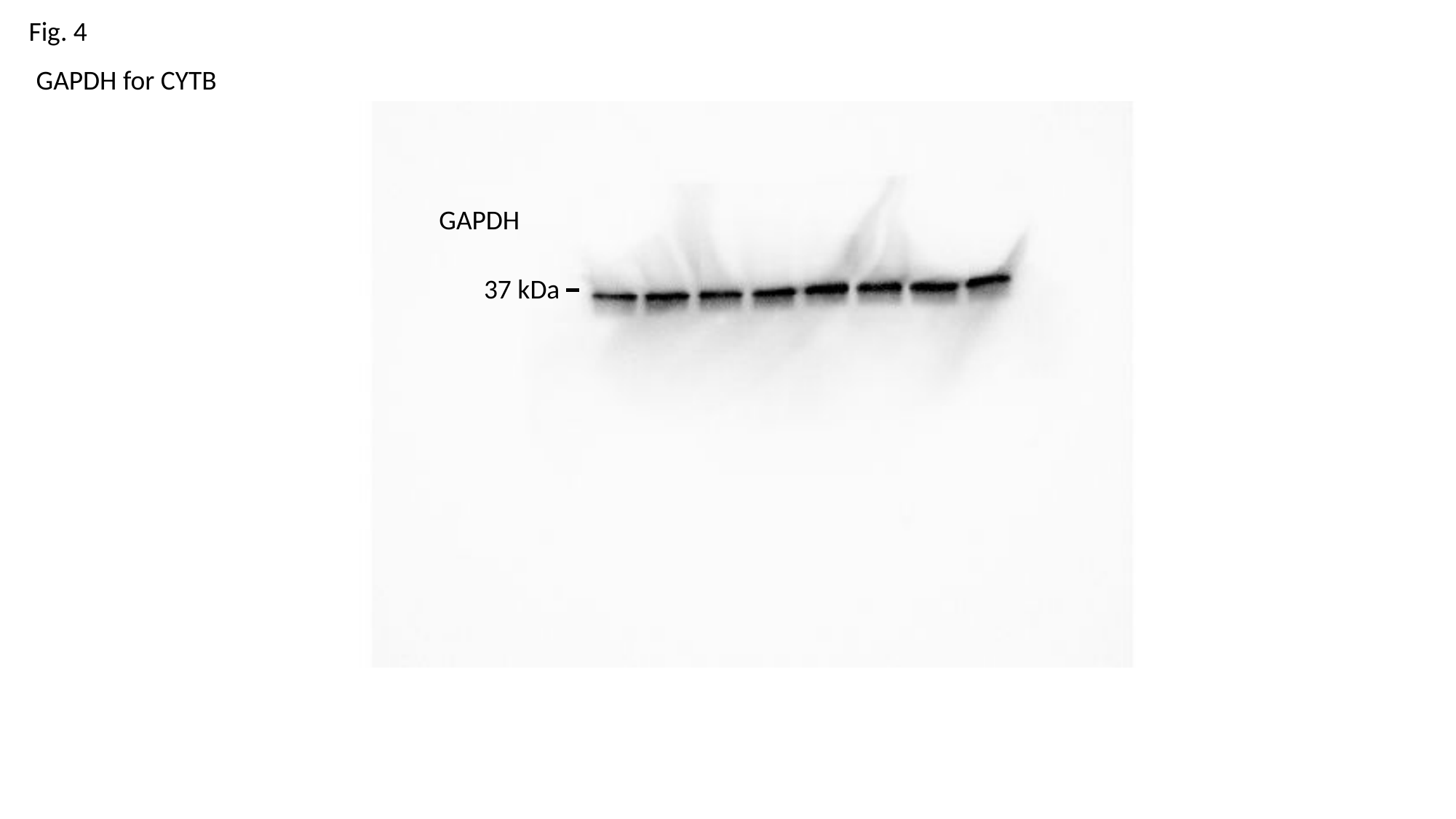

Fig. 4
GAPDH for CYTB
GAPDH
37 kDa
