# Supplementary figures and images for: Identification of a weight loss-associated causal eQTL in MTIF3 and the effects of MTIF3 deficiency on human adipocyte function
Source: eLife. 2023 Mar 6;12:e84168. doi: 10.7554/eLife.84168 (PMC10023155; doi:10.7554/eLife.84168)

## Slide 1
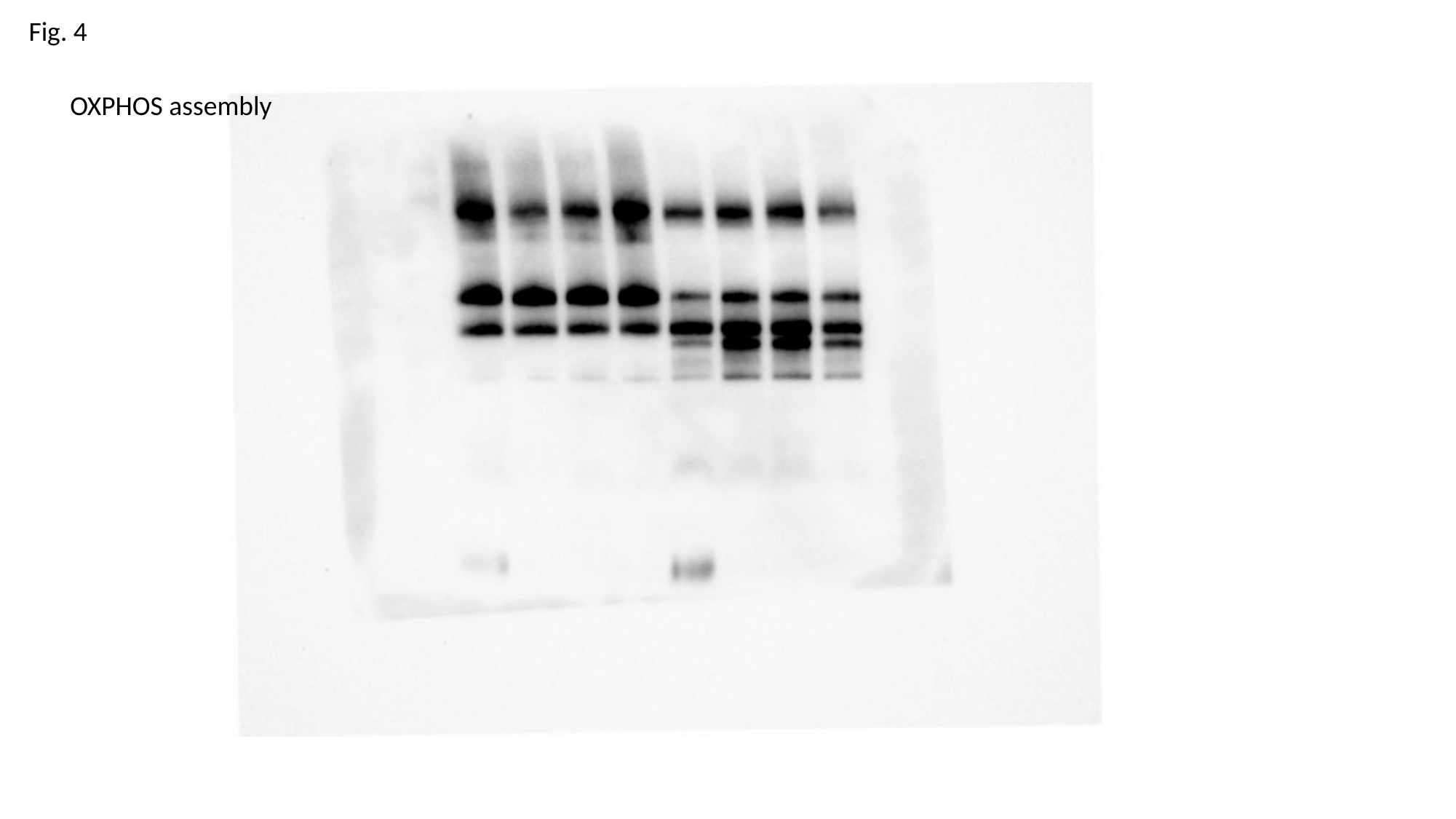

Fig. 4
OXPHOS assembly

Supplement: Figure 4—source data 2. [file elife-84168-fig4-data2.zip › Figure 4-source data 2.pptx]
