# Supplementary material for: Identification of a weight loss-associated causal eQTL in MTIF3 and the effects of MTIF3 deficiency on human adipocyte function
Source: eLife. 2023 Mar 6;12:e84168. doi: 10.7554/eLife.84168 (PMC10023155; doi:10.7554/eLife.84168)
Supplement: Supplementary file 1. — (a) Thirty-one SNPs in tight linkage disequilibrium (r2 ≥ 0.8) with the lead variant rs1885988 tiled down into 12 DNA segments of the MTIF3 gene for luciferase reporter assay. To fine map the transcriptional regulatory regions in the MTIF3 locus, we first identified the common genetic variants which were in tight linkage disequilibrium (r2 ≥ 0.8) with the lead variant rs1885988 in HaploReg v4.1. The identified 31 SNPs were tiled down into 12 DNA segments of the MTIF3 gene depending on PCR primer design constraints. (b) SNPs in MTIF3 locus associated with body mass index (BMI), whole-body fat mass and arm fat mass (right). We checked the rapid GWAS analysis results from 337,000 samples in the UK Biobank, which were made available by Benjamin Neale’s lab and visualized in Oxford BIG browser, we found SNPs in MTIF3 locus showed nominal associations with body weight-related traits including BMI, whole-body fat mass and arm fat mass (right). [file elife-84168-supp1.docx]

Supplementary file 1a

| **Rs ID** | **Position at hg38 chr13** | **Luciferase reporter construct** |
| --- | --- | --- |
| [rs7988412](https://pubs.broadinstitute.org/mammals/haploreg/detail_v4.1.php?query=&id=rs7988412) | 27426145 | 1 |
| [rs76790205](https://pubs.broadinstitute.org/mammals/haploreg/detail_v4.1.php?query=&id=rs76790205) | 27428748 | 2 |
| [rs10220056](https://pubs.broadinstitute.org/mammals/haploreg/detail_v4.1.php?query=&id=rs10220056) | 27429644 |  |
| [rs9581848](https://pubs.broadinstitute.org/mammals/haploreg/detail_v4.1.php?query=&id=rs9581848) | 27429860 |  |
| [rs9581849](https://pubs.broadinstitute.org/mammals/haploreg/detail_v4.1.php?query=&id=rs9581849) | 27431645 | 3 |
| [rs74183666](https://pubs.broadinstitute.org/mammals/haploreg/detail_v4.1.php?query=&id=rs74183666) | 27433628 | 4 |
| [rs7669](https://pubs.broadinstitute.org/mammals/haploreg/detail_v4.1.php?query=&id=rs7669) | 27435714 | 5 |
| [rs1885989](https://pubs.broadinstitute.org/mammals/haploreg/detail_v4.1.php?query=&id=rs1885989) | 27435980 | 6 |
| [rs1885988](https://pubs.broadinstitute.org/mammals/haploreg/detail_v4.1.php?query=&id=rs1885988) | 27436125 |  |
| [rs57724994](https://pubs.broadinstitute.org/mammals/haploreg/detail_v4.1.php?query=&id=rs57724994) | 27436252 |  |
| [rs76249173](https://pubs.broadinstitute.org/mammals/haploreg/detail_v4.1.php?query=&id=rs76249173) | 27436308 |  |
| [rs75439483](https://pubs.broadinstitute.org/mammals/haploreg/detail_v4.1.php?query=&id=rs75439483) | 27436375 |  |
| [rs77055070](https://pubs.broadinstitute.org/mammals/haploreg/detail_v4.1.php?query=&id=rs77055070) | 27436379 |  |
| [rs143190464](https://pubs.broadinstitute.org/mammals/haploreg/detail_v4.1.php?query=&id=rs143190464) | 27436414 |  |
| [rs74563672](https://pubs.broadinstitute.org/mammals/haploreg/detail_v4.1.php?query=&id=rs74563672) | 27436523 |  |
| [rs147240135](https://pubs.broadinstitute.org/mammals/haploreg/detail_v4.1.php?query=&id=rs147240135) | 27436782 |  |
| [rs12867531](https://pubs.broadinstitute.org/mammals/haploreg/detail_v4.1.php?query=&id=rs12867531) | 27436791 |  |
| [rs78334317](https://pubs.broadinstitute.org/mammals/haploreg/detail_v4.1.php?query=&id=rs78334317) | 27436817 |  |
| [rs76488435](https://pubs.broadinstitute.org/mammals/haploreg/detail_v4.1.php?query=&id=rs76488435) | 27436853 |  |
| [rs79023532](https://pubs.broadinstitute.org/mammals/haploreg/detail_v4.1.php?query=&id=rs79023532) | 27436942 |  |
| [rs74811644](https://pubs.broadinstitute.org/mammals/haploreg/detail_v4.1.php?query=&id=rs74811644) | 27437021 |  |
| [rs45622135](https://pubs.broadinstitute.org/mammals/haploreg/detail_v4.1.php?query=&id=rs45622135) | 27437457 | 7 |
| [rs9581850](https://pubs.broadinstitute.org/mammals/haploreg/detail_v4.1.php?query=&id=rs9581850) | 27438128 |  |
| [rs12018313](https://pubs.broadinstitute.org/mammals/haploreg/detail_v4.1.php?query=&id=rs12018313) | 27438129 |  |
| [rs139686876](https://pubs.broadinstitute.org/mammals/haploreg/detail_v4.1.php?query=&id=rs139686876) | 27438919 | 8 |
| [rs9581852](https://pubs.broadinstitute.org/mammals/haploreg/detail_v4.1.php?query=&id=rs9581852) | 27439611 |  |
| [rs9579083](https://pubs.broadinstitute.org/mammals/haploreg/detail_v4.1.php?query=&id=rs9579083) | 27443133 | 9 |
| [rs9581854](https://pubs.broadinstitute.org/mammals/haploreg/detail_v4.1.php?query=&id=rs9581854) | 27443645 | 10 |
| [rs9581855](https://pubs.broadinstitute.org/mammals/haploreg/detail_v4.1.php?query=&id=rs9581855) | 27443877 |  |
| [rs67785913](https://pubs.broadinstitute.org/mammals/haploreg/detail_v4.1.php?query=&id=rs67785913) | 27451171 | 11 |
| [rs9512699](https://pubs.broadinstitute.org/mammals/haploreg/detail_v4.1.php?query=&id=rs9512699) | 27455759 | 12 |

Supplementary file 1b

| Phenotype | CHR | BP | SNP | Ref | Alt | P-value | Beta | MAF |
| --- | --- | --- | --- | --- | --- | --- | --- | --- |
| BMI | 13 | 28010734 | rs1218824 | A | G | 3.1x10^-11 | 0.017 | 34% |
|  | 13 | 28011963 | rs1218822 | G | A | 3.0x10^-11 | 0.017 | 34% |
|  | 13 | 28012527 | rs9512696 | G | A | 3.1x10^-11 | 0.017 | 34% |
|  | 13 | 28013501 | rs7992832 | T | C | 7.0x10^-11 | -0.018 | 28% |
|  | 13 | 28018237 | rs9507895 | C | G | 7.20x10^-11 | -0.018 | 28% |
|  | 13 | 28020180 | rs4771122 | A | G | 1.70x10^-10 | -0.017 | 23% |
|  | 13 | 28022914 | rs4771123 | T | C | 1.30x10^-10 | -0.017 | 28% |
|  | 13 | 28033485 | rs9512702 | C | T | 1.0x10^-9 | -0.017 | 26% |
|  | 13 | 28036062 | rs1967772 | A | G | 5.50x10^-11 | -0.018 | 29% |
| Whole body fat mass | 13 | 28010734 | rs1218824 | A | G | 3.1x10^-11 | 0.017 | 34% |
|  | 13 | 28011963 | rs1218822 | G | A | 3.0 x10^-11 | 0.017 | 34% |
|  | 13 | 28012527 | rs9512696 | G | A | 3.1x10^-11 | 0.017 | 34% |
|  | 13 | 28013501 | rs7992832 | T | C | 7.0x10^-11 | -0.018 | 28% |
|  | 13 | 28018237 | rs9507895 | C | G | 7.20x10^-11 | -0.018 | 28% |
|  | 13 | 28020180 | rs4771122 | A | G | 1.70x10^-10 | -0.017 | 23% |
|  | 13 | 28022914 | rs4771123 | T | C | 1.30x10^-10 | -0.017 | 28% |
|  | 13 | 28036062 | rs19967772 | A | G | 5.50x10^-11 | -0.018 | 29% |
|  | 13 | 28049176 | rs1007700 | T | C | 7.30x10^-9 | 0.015 | 29% |
|  | 13 | 28056547 | rs9507906 | G | C | 7.80x10^-9 | -0.015 | 29% |
| Arm fat mass (right) | 13 | 28010734 | rs1218824 | A | G | 3.1x10^-11 | 0.017 | 34% |
|  | 13 | 28011963 | rs1218822 | G | A | 3.0x10^-11 | 0.017 | 34% |
|  | 13 | 28012527 | rs9512696 | G | A | 3.1x10^-11 | 0.017 | 34% |
|  | 13 | 28013501 | rs7992832 | T | C | 7.0x10^-11 | -0.018 | 28% |
|  | 13 | 28018237 | rs9507895 | C | G | 7.20x10^-11 | -0.018 | 28% |
|  | 13 | 28018370 | rs4771121 | A | G | 1.20x10^-9 | -0.016 | 28% |
|  | 13 | 28022914 | rs4771123 | T | C | 1.30x10^-10 | -0.017 | 28% |
|  | 13 | 28033485 | rs9512702 | C | T | 1.0x10^-9 | -0.017 | 26% |
|  | 13 | 28036062 | rs1967772 | A | G | 5.50x10^-11 | -0.018 | 29% |
|  | 13 | 28049176 | rs1007700 | T | C | 3x10^-9 | 0.016 | 29% |
|  | 13 | 28049143 | rs1007701 | G | C | 8.5x10^-9 | 0.016 | 26% |
|  | 13 | 28049506 | rs9507904 | T | C | 1.2x10^-8 | -0.016 | 26% |
